# Global Standardization of Lipid/Lipoprotein Testing

**DOI:** 10.14789/jmj.JMJ24-0019-P

**Published:** 2024-09-30

**Authors:** TAKASHI MIIDA

**Affiliations:** 1 Department of Clinical Laboratory Medicine, Juntendo University Graduate School of Medicine, Tokyo, Japan; 1 Department of Clinical Laboratory Medicine, Juntendo University Graduate School of Medicine, Tokyo, Japan

**Keywords:** reference material, traceability, biomarker, standardization, cardiovascular disease

## Abstract

As lipid/lipoprotein research elucidated the mechanism of atherosclerosis, lipid/lipoprotein tests were developed for simple and rapid screening, diagnosis, and management of dyslipidemic patients. In 1988, the Centers of Disease Control and Prevention (CDC) in the United States initiated a global standardization program for lipid/lipoprotein testing through the Cholesterol Reference Measurement Laboratory Network (CRMLN), in which the chemical lipid measurements were adopted as reference measurement procedures (RMPs). In 2010, an American group questioned the accuracy of the direct LDL-C and HDL-C assays, causing a great deal of confusion. Our two comparative studies evaluating the direct LDL-C and HDL-C assays have removed reagents with poor analytical performance from the market and demonstrated that the assays are currently accurate enough for clinical use. Because these traditional chemical methods require a high level of technical expertise, RMP are shifting from chemical reaction-based methods to mass spectrometry-based methods. We are now working on the standardization of lipoprotein(a) using the mass spectrometry-based method as an RMP.

## Introduction

In clinical practice, serum lipid and lipoprotein testing is used as a routine clinical laboratory test to assess the risk of atherosclerotic disease. The important role of dyslipidemia in the pathogenesis of atherosclerosis is supported by a large number of experimental, epidemiological and clinical studies^1-5)^. Currently, most lipids and lipoproteins are measured in individual clinical laboratories using high-performance autoanalyzers. Historically, the development of new reagents has been linked to advances in lipoprotein research. To ensure the clinical utility of lipid/lipoprotein assays as risk markers, we continue to pursue global standardization efforts on a worldwide scale.

In this review, we will first focus on the relationship between advances in lipid/lipoprotein research and the development of clinical laboratory tests. I will then summarize our studies that have contributed to the standardization of direct LDL-C and HDL-C methods. In the last part, I will describe a recent topic of standardization of lipid/lipoprotein assays.

## A history of lipid/lipoprotein research and assay development

As researchers have uncovered the important role of cholesterol and lipoproteins in the development and progression of atherosclerosis, the need for lipid/lipoprotein measurement has increased. Cholesterol research and development of cholesterol assays was slow until the 1800s. In the early 1900s, the relationship between atherosclerosis and cholesterol was clarified. At the same time, lipoproteins such as HDL and LDL were identified in serum (Table 1). In 1948, the Framingham Study, a large community-based cohort study, was initiated. At that time, no one had established methods for quantifying lipoproteins. The first report of the Framingham Study was published in 1957, showing that total cholesterol (TC) concentration was associated with the risk of coronary heart disease^6)^. It is reasonable that they used TC as a risk marker because they could not measure LDL cholesterol (LDL-C) and HDL-C at recruitment. From the 1950s to the 1970s, many researchers published methods to separate lipoproteins by ultracentrifugation. In 1972, Friedewald reported a formula for estimating LDL-C from TC, triglycerides, and HDL-C^7)^. Although this equation is now used worldwide, it took several years before it was widely used in clinical practice. The Lipid Research Clinics-Coronary Primary Prevention Trial (LRC- CPPT) is a landmark study in lipoprotein research. It began the year after Fiedewald’s paper was published. They gave the primary prevention group the cholesterol-lowering drug cholestyramine (an anion exchange resin) and tested whether it reduces the incidence of coronary heart disease. In this study, LDL was separated by a combination of ultracentrifugation and precipitation, and cholesterol was measured by the Abell-Kendall method, a traditional chemical method for quantifying cholesterol. The enzymatic method for TC was developed in 1975, 2 years after the start of the LRC-CPPT. Therefore, it is not surprising that the chemical method was used to measure LDL-C concentration in the LRC-CPPT. The enzymatic method soon replaced the chemical method for measuring cholesterol in clinical laboratories around the world. It should be noted that statin was discovered at that time by a Japanese scientist. Statin is a powerful cholesterol-lowering agent and is used as a first-line treatment for hypercholesterolemia. However, it took 26 years for statins to become available for clinical use in Japan.

In 1988, the Centers for Disease Control and Prevention (CDC) initiated a worldwide lipid standardization program with the Cholesterol Reference Method Laboratory Network (CRMLN). Japan has been a member of this network from the very beginning. The CDC/ CRMLN uses the beta- quantification (BQ) method as the reference measurement procedure (RMP) for LDL-C. Since the BQ method is based on the LRC-CPPT method, the Abell-Kendall method was adopted for cholesterol quantification. At the time, there were reports of methods to quantify cholesterol using mass spectrometry. However, the Framingham Study (an epidemiological study) and the LRC-CPTPT (a drug intervention study) had used the Abell-Kendall method to measure cholesterol levels. To maintain continuity of cholesterol levels, the CDC/CRMLN adopted the BQ method (which uses the Abell-Kendall method for cholesterol measurement) for their lipid standardization programs. Therefore, it is not surprising that both the 4S (the secondary prevention trial published in 1994)^8)^ and the WOS- COPS (the primary prevention trial published in 1995)^9)^ used the LRC-CPPT method to measure LDL-C levels. As Friedewald’s equation became more widely accepted, it was increasingly used in subsequent drug intervention trials because of its simplicity. The method of LDL-C measurement is often not reported in papers on epidemiologic studies or drug intervention trials. Researchers should be aware of the importance of lipid/lipoprotein measurement methods in their studies.

**Table 1:** Chronology of cholesterol/lipoprotein studies and cholesterol/lipoprotein assay development

Year	Cholesterol/lipoprotein studies	Cholesterol/lipoprotein assay development
1800's		
1856	• Massive cholesterol deposits in atherosclerotic lesions (Virchow, et al.)	
1885		• Liebermann-Burchard reaction (for measuring cholesterol)
Early 1900's		
1913	• High cholesterol diet induced atherosclerosis in rabbits (Antischkow, et al.)	
1929		• Discovery of HDL (Macheboeuf, et al.)
1947		• Discovery of LDL (Oncley, et al.)
1948	• Initiation of the Framingham Heart Study	
1949		• Establishment of lipoprotein fractionation by ultracentrifugation (Gofman, et al.)
Late 1900's		
1957	• Hypercholesterolemia found to be a risk factor for coronary heart disease	
1972		• LDL-C calculation method (Friedewald, et al.)
1973	• Discovery of compactin (Endo, et al.) • Initiation of the Lipid Research Clinics-Coronary Primary Prevention Trial (LRC-CPPT)	• Development and dissemination of the enzymatic method for measuring total cholesterol
1984	• Cholestyramine reduced ischemic heart disease (LRC-CPPT)	
1988		• CDC launched Cholesterol Reference Measurement Laboratory Network (CRMLN) for global lipid standardization
1989	• Pravastatin launched in Japan	
1994	• Simvastatin reduced mortality and morbidity in the secondary prevention group (4S).	• Direct HDL-C assay launched
1995	• Pravastatin reduced mortality and morbidity in the primary prevention group (WOS-COP)	
1998		• Direct LDL-C assay launched
2000's		
2002		• Koichi Tanaka received Nobel Prize in Chemistry for mass spectrometry
2007	• Hypercholesterolemia is diagnosed by LDL-C (JAS 2007 guideline)	
2010	• Direct LDL-C and HDL-C assays showed poor analytical performance in the diseased group (Miller)	
2011		• CDC reported the reference measurement procedure (RMP) for cholesterol using mass spectrometry.
2012	• Friedewald's equation should be used for LDL-C determination (JAS 2012 guideline) • Comparative study of direct LDL-C assays published (Miida, et al.)	
2014	• Comparative study of direct HDL-C assays published (Miida, et al.)	
2017	• Accuracy of direct LDL-C assays in postprandial samples confirmed (Miida , et al) • Use of direct LDL-C assays approved (JAS 2017 guideline)	

JAS, Japan Atherosclerosis Society

## Development of direct LDL-C assays and their reliability

Because it takes several days to separate HDL by sequential ultracentrifugation, the precipitation method was developed for the measurement of HDL-C. In this method, apolipoprotein B-containing lipoproteins are precipitated with polyanions and divalent cations and removed by centrifugation. The cholesterol concentration in the upper fraction was measured by an enzymatic method. However, because of the need for pretreatment, it is difficult to process large numbers of samples using this method. In addition, the calculation of LDL-C by Friedewald’s formula requires the concentration of HDL-C. Furthermore, Friedewald’s formula cannot be used when subjects have fasting triglycerides > 400 mg/dL or are in the postprandial state. Under these circumstances, the direct method of HDL-C was first reported in 1995^10)^. Because it requires no pretreatment, this method was quickly adopted in clinical laboratories worldwide. In the 1990s, the direct LDL-C method was also developed by several Japanese manufacturers. Unlike the direct HDL-C method, the direct LDL-C method was not as widely used at the time of its introduction, probably because LDL-C could be determined using the Friedewald equation. In addition, the Japanese health insurance system reimburses only 2 out of 3 items when TC, LDL-C, and HDL-C are measured simultaneously. However, 2 factors have promoted the spread of the direct LDL-C method. First, the revision of the Japanese guidelines in 2007 changed their recommendation that hypercholesterolemia should be diagnosed based on LDL-C rather than TC levels. Second, nationwide “Lifestyle Health Check-ups and Health Guidance” began in 2008. In this Japanese national project, LDL-C is selected as one of the mandatory tests. The number of sites using the direct LDL-C method has gradually increased.

As the direct method became routinely used in laboratories, inter-reagent discrepancies in LDL-C were reported in special conditions (i.e., cholestatic liver disease, familial type III hyperlipidemia, hyper- alpha-lipoproteinemia, etc.). Shortly after my appointment at Juntendo University, Miller et al. compared LDL-C results between 7 direct assays and the BQ method using fresh plasma and reported in 2010 that the analytical performance of the direct LDL-C methods was remarkably poor with most reagents, especially when used in the diseased group^11)^. This paper caused a great deal of confusion in clinical and health checkup settings. As a result, the 2012 edition of the Japanese guidelines states that the Friedewald’s equation should be used for LDL-C measurement^12)^.

**Figure 1:**
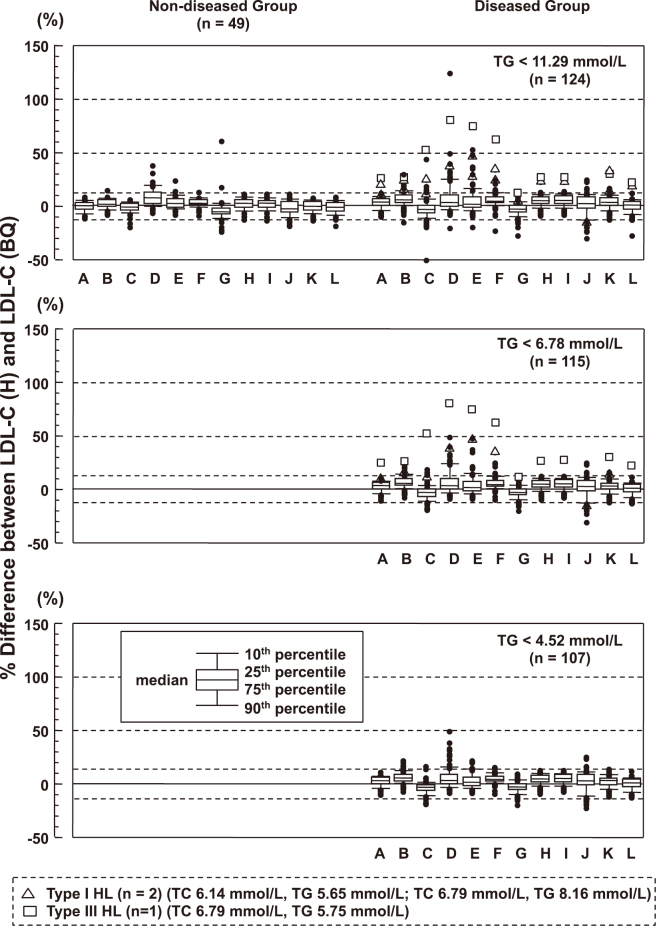
Box-and-wisker plots of the % difference between the LDL-C (H) and LDL-C (BQ) for non-diseased and diseased groups LDL-C (H), LDL-C measured by the homogeneous assay (the direct assay); LDL- (BQ), LDL-C measured by the BQ method. Percent differences were calculated by the following equation: [LDL-C (H)−LDL-C (BQ)]×100/LDL-C (BQ). A, Denka Seiken; B, Wako; C, Sysmex; D, Serotec; E, Fureiya; F, Kyowa; G, Toyobo; H, Shino-Test; I, Sekisui-Medical; J, Ortho Clinical Diagnostics; K, Siemens Healthcare Diagnostics; L, Beckman Coulter. The National Cholesterol Education Program (NCEP) requires the total error (TE) to be less than 13% for both LDL-C and HDL-C. (Reproduced from reference #^13^: Miida et al. Atherosclerosis 2012)

**Figure 2:**
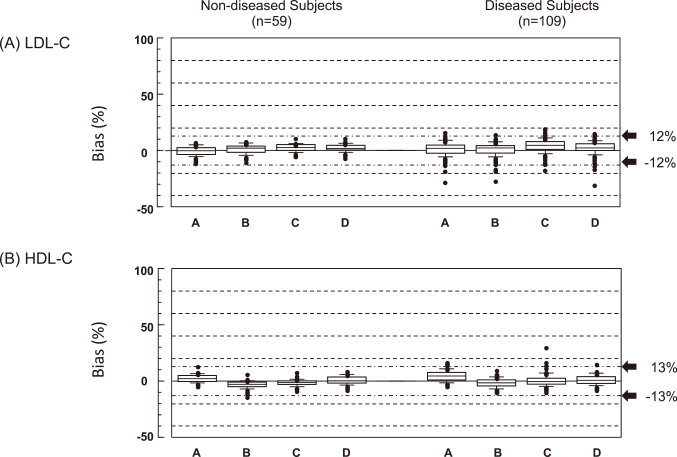
Box-and-whisker plots of the %bias of the direct LDL-C and HDL-C assays (HAs) in the non-diseased and diseased groups The LDL-C and HDL-C concentrations of fresh blood samples were simultaneously measured using the direct assays and the reference measurement procedures (RMPs) of the Centers for Disease Control and Prevention (CDC). %bias is the percentage deviation of the direct method value from the RMP value. A, Denka Seiken; B, Wako; C, Kyowa Medex; D, Sekisui Medical. The National Cholesterol Education Program (NCEP) requires the total error (TE) to be less than 12% for LDL-C, and 13% and HDL-C. (Reproduced from reference #^15^: Miida, et al. J Atheroscler Thromb 2017)

After reading the Miller’s paper in detail, we have found a number of problems with their study. For example, they enrolled some blood samples from the very rare patients with primary dyslipidemia whose lipoprotein composition was significantly different from normal. Therefore, the Japan Atherosclerosis Society, in collaboration with the Japan Society of Clinical Chemistry, the Japanese Society of Laboratory Medicine, and the Japan Association of Clinical Reagents Industries, conducted a multicenter collaborative study to determine whether the direct LDL-C reagents could accurately measure LDL-C in healthy subjects and patients with common diseases encountered in routine medical care. We found that LDL-C measured by most direct methods was in good agreement with that measured by the BQ method in the non-diseased group. However, LDL-C measured by some direct methods was higher than that measured by the BQ method (Figure 1)^13)^. As the manufacturers of the reagents with poor analytical performance voluntarily stopped selling their reagents, improved their reagents, or changed the assigned values of their calibrators, only 4 companies remained to sell their own LDL-C direct methods. The other companies eventually adopted and sold the good reagents from these 4 companies to their customers. We did a similar study of the direct HDL-C assays^14)^. We conducted a multicenter study again and confirmed that the direct LDL-C and HDL-C method reagents commercially available in Japan maintained accuracy (Figure 2)^15)^. At that time, some insisted that LDL-C should be excluded form the mandatory component of the Specific Health Screening. The paper was published in 2017, and supported the use of the direct LDL-C method in the Specific Health Screening. The 2017 edition of the Japanese guidelines also endorsed the use of the direct LDL-C method in the diagnosis and management of hypercholesterolemia^16)^. Recent foreign guidelines approve the use of the direct LDL-C method along with apolipoprotein B in cases of low LDL-C for which the Friedewald’s equation is considered inaccurate^17, 18)^. The 2022 edition of the Japanese guidelines allows postprandial blood samples for screening of dyslipidemia^19)^. Since the direct LDL-C method is as accurate in the postprandial state as in the fasting state, the opportunity to use the direct LDL-C method will increase.

## Mass spectrometry-based RMP for the global standardization

As I mentioned earlier, the standardization of lipid testing in Japan is being done in conjunction with the CDC/CRMLN. The Abell-Kendall method is being used as the reference method for measuring total cholesterol, LDL-C, and HDL-C. The CDC provides another program for TC using isotope dilution mass spectrometry (ID/MS)^20)^. On the other hand, the LDL-C and HDL-C programs still use the Abell-Kendall method to determine cholesterol. The Abell-Kendall method is not only time- consuming, but also requires a high level of technician-dependent laboratory skills. We are afraid that there will be no successor in the near future. Due to interference with phytosterols and cholesterol precursors, serum cholesterol concentrations measured by the Abell-Kendall method are approximately 2% higher than those measured by ID/MS. The full transition to ID/MS in the LDL-C and HDL-C programs has not yet been achieved due to the lack of actual data on the difference in the LDL and HDL fractions between these two methods. For a smooth transition to ID/MS, we should conduct the comparative study in Japan. After that, it is essential to reach a consensus with experts in epidemiology and clinical medicine.

## Global standardization of Lp(a)

Lipoprotein(a) [Lp(a)] is a complex of LDL and apoliprotein(a) [apo(a)] and is recognized as an independent risk factor for cardiovascular events. The apo(a) of Lp(a) is covalently linked to the apoB of LDL. Apo(a) is divided into many isoforms, which are extremely heterogeneous in size due to the variable number of Klingle IV type 2 repeats^21)^. Because the recognition sites of apo(a) differ among the antibodies of the immunoassays, there is a large variation in Lp(a) values between them. To address this problem, a working group was established by the World Health Organization (WHO) and the International Federation of Clinical Chemistry (IFCC) in the 1990s. The working group produced the serum-based reference material called SRM2B^22)^, which was validated by the isoform-independent enzyme-linked immunoassay. Although SRM2B was distributed to manufacturers, global standardization of Lp(a) was ultimately not achieved^23)^.

Recent advances in drug development have changed this situation. While existing drugs lower Lp(a) by up to 20%, new nucleic acid drugs lower Lp(a) by 80-90%^24)^. These drugs are expected to be available for clinical use within 3 to 6 years. Therefore, there is a growing need for international standardization of Lp(a). Last year, the IFCC reported and endorsed the RMP for Lp(a) using mass spectrometry^25)^. This method is apo(a) isoform- independent. Lp(a) concentration is expressed in the SI unit, which is metrologically correct. We have been working on the Lp(a) project since last year. In the first study, we obtained the panel sera from CDC. Their Lp(a) values were determined by the IFCC-endorsed mass spectrometry. The results strongly suggest that we can achieve Lp(a) standardization. The second study is underway to evaluate a larger number of reagents. Global Lp(a) standardization is becoming a reality.

## Conclusions

In order to properly manage dyslipidemia and reduce the risk of cardiovascular disease, global standardization of lipid/lipoprotein testing is essential. Mass spectrometry is a powerful tool to achieve this. We will continue to contribute to the global standardization of lipid/lipoprotein testing through our activities.

## Funding

No funding was received.

## Author contributions

TM wrote this manuscript.

## Conflicts of interest statement

The author has no conflict of interest to disclose.
